# The integration of occupational therapy into primary care: a multiple case study design

**DOI:** 10.1186/1471-2296-14-60

**Published:** 2013-05-16

**Authors:** Catherine Donnelly, Christie Brenchley, Candace Crawford, Lori Letts

**Affiliations:** 1 School of Rehabilitation Therapy, Queen’s University, 31 George Street, Kingston, ON, K7L 3N6, Canada; 2 Faculty of Education, Queen’s University, 31 George Street, Kingston, ON, K7L 3N6, Canada; 3 Ontario Society of Occupational Therapists, 55 Eglinton Ave. E., Suite 210, Toronto, ON, M4P 1G8, Canada; 4 Wise Elephant Family Health Team, 280 Main Street North, Brampton, ON, L6V 1P6, Canada; 5 School of Rehabilitation Science, McMaster University, 1400 Main St. W, Hamilton, ON, L8S 1C7, Canada

**Keywords:** Inteprofessional primary care, Collaboration, Family health teams, Multiple case study design, Occupational therapy, Integration

## Abstract

**Background:**

For over two decades occupational therapists have been encouraged to enhance their roles within primary care and focus on health promotion and prevention activities. While there is a clear fit between occupational therapy and primary care, there have been few practice examples, despite a growing body of evidence to support the role. In 2010, the province of Ontario, Canada provided funding to include occupational therapists as members of Family Health Teams, an interprofessional model of primary care. The integration of occupational therapists into this model of primary care is one of the first large scale initiatives of its kind in North America. The objective of the study was to examine how occupational therapy services are being integrated into primary care teams and understand the structures supporting the integration.

**Methods:**

A multiple case study design was used to provide an in-depth description of the integration of occupational therapy. Four Family Health Teams with occupational therapists as part of the team were identified. Data collection included in-depth interviews, document analyses, and questionnaires.

**Results:**

Each Family Health Team had a unique organizational structure that contributed to the integration of occupational therapy. Communication, trust and understanding of occupational therapy were key elements in the integration of occupational therapy into Family Health Teams, and were supported by a number of strategies including co-location, electronic medical records and team meetings. An understanding of occupational therapy was critical for integration into the team and physicians were less likely to understand the occupational therapy role than other health providers.

**Conclusion:**

With an increased emphasis on interprofessional primary care, new professions will be integrated into primary healthcare teams. The study found that explicit strategies and structures are required to facilitate the integration of a new professional group. An understanding of professional roles, trust and communication are foundations for interprofessional collaborative practice.

## Background

There is a clear fit between occupational therapy (OT) and primary care. Both view health in a holistic manner and seek to support individuals and communities in achieving and maintaining a healthy lifestyle [1,2]. While there is evidence to support the role of occupational therapy in health promotion and prevention, there have been few practice examples of occupational therapy within primary care settings [3,4].

The lack of an occupational therapy presence in primary care can be attributed to a number of factors [5]. First and foremost, there has not been funding for occupational therapy in primary care, both in Canada and internationally [5]. Second, primary care has traditionally been delivered in solo practitioner models [6]. Finally, the occupational therapy profession has traditionally focused on the rehabilitation or remediation of function versus health promotion [7].

In 2003, the First Ministers of Canada committed to ensuring that half of Canadians would have access to multidisciplinary primary care teams by 2011 [8]. While this has not yet been achieved, the province of Ontario’s commitment to health reform has resulted in the establishment of Family Health Teams, an innovative model of interprofessional primary care [9]. There are currently 200 teams that serve approximately 25% of the province’s population.

Each Family Health Team is interprofessional in nature; however there is considerable variability in structure, size and organizational dimensions. A Family Health Team may consist of a single site or may be comprised of multiple offices that have common programs or structures such as an electronic medical record (EMR), programs and management. The complement of interdisciplinary health professionals also varies according to the specific needs of the community.

While the initial list of funded interdisciplinary health providers did not include occupational therapists, in March 2009 the Ontario government committed funds to include occupational therapy services in Family Health Teams [10]. At the initiation of the study, 20 teams had occupational therapists within their team complement. Ontario’s initiative is one of the first examples of large-scale integration of occupational therapy into primary care teams in North America.

A growing number of national and international studies have documented the structures and processes to support interprofessional primary care teams [11,12]. However, few of these studies have included occupational therapy within the team complement and no study has exclusively examined the implementation of occupational therapy into a new or existing primary care team.

A handful of articles have examined the integration of other professionals into primary care teams [13-15]. While these findings might provide insights for occupational therapy, each profession entering primary care will have unique features and support the team through unique roles. Occupational therapists have a long history in working in team- based environments and therefore the implementation of occupational therapy services may be experienced differently than professions that have been primarily consultative.

Interprofessional teams are poised to play a greater role in the delivery of primary care in Canada and abroad [16,17]. It is anticipated that more disciplines will continue to enter primary care, making it critical to understand how professionals are being introduced into primary care teams. The purpose of the paper is to examine how occupational therapy is being integrated into primary care teams and understand the structures and processes supporting the integration.

## Methods

The study aimed to explore the primary guiding question: What structures and processes support the integration of occupational therapy in Family Health Teams? A multiple case study design [18] was conducted that included four Family Health Team sites within the province of Ontario, Canada. Case study research seeks to investigate real life experiences within the context in which it occurs and involves the collection of detailed information using a variety of data collection methods [18-20]. As there are few documented examples of occupational therapists in primary care, a case study design enabled an in-depth exploration of how occupational therapy was being integrated into interprofessional primary care teams. As per case study methodology as outlined by Yin [18], each case provided an opportunity for the replication of the outlined questions and methods.

### Site identification

Four cases (Family Health Teams) were identified from the approximately 20 that employed occupational therapists at the time of the study. The sites were chosen to reflect different dimensions of service provision that may influence the role and integration of occupational therapy. The literature on interprofessional collaborative practice has identified certain elements that support interprofessional collaborative care, including: (1) EMR, (2) team size, and (3) co-location of health professionals [6,13]. Each dimension was considered in the identification of the cases. Two further dimensions were considered in the case selection; academic versus community and rural versus urban. While there is little evidence examining the role of occupational therapist in primary care, the literature has described occupational therapy working with a wide range of client populations and conditions [4]. Therefore the nature and duration of clinical experience of occupational therapist as well as the full-time equivalency (FTE) were also thought to be important elements to consider in the identification of cases. Purposeful sampling of sites was used with the intent to sample breadth of communities, teams, and occupational therapists.

### Participants

Information letters were sent to the Executive Director at each site describing the study and seeking approval for participation. All occupational therapists working at the Family Health Teams were asked to participate. The Executive Director and the lead physician were also invited due to their leadership and decision making roles on the team. In addition, any member of the team that provided collaborative patient care with the occupational therapist was also considered to be eligible for the study. The occupational therapist(s) at each Family Health Team acted as the main contact for liaising and coordinating interviews with the staff.

Ethics approval was provided by Queen’s University Health Sciences Research Ethics Board.

### Data collection

Data collection drew on multiple forms of evidence including semi-structured interviews, document analyses and questionnaires. The principal investigator (CD) visited each Family Health Team to retrieve documents for analyses, distribute questionnaires and conduct interviews with key informants. See Table 1 for list of disciplines interviewed at each site. All interviews were conducted between the February-May 2012 using a semi-structured interview guide. Questions were developed by the research team and were informed by the literature on interprofessional collaborative primary care [11,12]. Questions fell under five broad categories including; roles (how would you describe your role, how did you establish your role), physical space (i.e. location of team members and primary care sites), community collaborations, collaborative practice (i.e. nature, processes and structures to support collaborative practice) and processes (i.e. nature and use of electronic medical record). Additional questions regarding funding for occupational therapy were included in the interview guide for the Executive Director and questions related to clinical practice were removed.

**Table 1:** Interview summary

	**Case 1**	**Case 2**	**Case 3**	**Case 4**
**ED**	1	1	1	1
**OT**	2	1	1	2
**Nurse Practitioner**		1		1
**Social Worker**	2	1	1	1
**Physician**	1	1	1	2
**Dietician**			1	
**Diabetes Educator**	1			

Program documents included job descriptions, occupational therapy assessments, team mission and vision. The web pages of each Family Health Team were viewed to obtain further information about team collaboration, and sites were contacted if further questions about the nature of occupational therapy services were identified. Two sites were contacted to clarify demographic information (number of sites and number of physicians) and the occupational therapist(s) at each site was contacted to provide further details on the referral process to occupational therapy. A Family Health Team Profile was completed by each Executive Director to obtain descriptive information about the Family Health Team demographics, including the type of electronic medical record system, number of rostered patients and health professional make-up. An Occupational Therapy Profile was completed by each occupational therapist to obtain information about their educational background and work experiences.

### Data analyses

Both within-case and cross-case analyses were conducted [18,19]. Pattern matching was then used as the overall analytic strategy. This approach “compares an empirically based pattern with a predicted one” [18, p 106], where propositions are developed prior to data collection in order to identify a predicted pattern of variables. Propositions for this study were derived from the literature on interprofessional collaborative practice. A number of factors have been found to support interprofesional practice. One of these is the extent to which there is a shared understanding of team members’ roles and scopes of practice [12]. This was felt to be particularly relevant for the study as occupational therapists were new professionals within the teams. Studies have also identified the nature of team processes and organizational structures to be important influences on collaboration, and the nature of team processes was anticipated to influence the integration of occupational therapy [21]. The use of electronic medical records (EMR) have become standard in Family Health Teams in Ontario, Canada [22] and have already been found to support internal communication. Occupational therapists’ access and use of EMRs thus become an important element to consider [13]. Therefore, the two study propositions were:

1. Integration of occupational therapy into the Family Health Team will depend on the understanding of the occupational therapy role by team members, and structures to support interprofessional collaborative practice.

2. The EMR will be pivotal in supporting the integration of occupational therapy.

Each case was first analyzed individually, followed by cross-site analyses to determine common themes [19]. Data obtained from documents were extracted using apriori document analysis forms. Tables and matrixes were used to visually examine the data for each case and across cases. Qualitative interview data were digitally recorded and transcribed verbatim by a research assistant. Atlas ti, a qualitative data analysis and research software program, was used to code data and identify themes both within and across cases. All transcripts were read and re-read by the primary author and preliminary codes were established. A number of strategies were used to establish trustworthiness [23,24]. Four transcripts were read and independently coded by a second investigator (LL) using the preliminary coding structure. Transcripts were selected from four different health professions to ensure the coding structure could be applied across transcripts. Any discrepancies in coding were noted and discussed until consensus was reached. Two revisions to the coding structure were made; the first involved collapsing two codes into one code, the second revision involved renaming a code to better reflect the essence of the statements being captured.

A second strategy to establish trustworthiness involved member checking. Occupational therapists were provided with a preliminary summary of their site and asked to contact the primary author if any errors were noted, or if additional information should be included. None of the participants reported any errors or provided further information.

A third strategy involved triangulation of data methods, sources and investigators. The study included a number of data methods including interviews, questionnaires and document analyses. Each contributed to the understanding of how occupational therapists are integrated into primary care and structures to support the integration. Participants included members from a range of disciplines across four sites in order to provide different perspectives and experiences on the integration of occupational therapists. Finally, the investigation team was made up four occupational therapists; two academics (CD, LL), one administrative (CB) and one clinician working in primary care (CC). The diversity of the team brought unique perspectives to the design, implementation and analyses and grounded the study in both research and practice.

## Results

Table 2 provides a description of the four sites. Patient rosters ranged from 7,200 to 42,000 patients and sites were located in both rural and urban centres. Three sites were community sites and one was an academic site. The academic site had a dual mandate to provide both primary care services, and to educate medical students/residents and other health disciplines. Occupational therapists were all relatively new to their positions with a range of 3 to 18 months. Occupational therapists in two sites had less than five years experience, while two sites had occupational therapists with 15 and more years of experience. Each site had a unique complement of health providers, which included: chiropodists, psychologists, social workers, dieticians, physician assistants, pharmacists, patient educators, mental health workers, health promoters, respiratory therapists, case managers, nurses, nurse practitioners, and physicians.

**Table 2:** Site profiles

	**Case 1**	**Case 2**	**Case 3**	**Case 4**
**Urban/rural**	Rural	Urban	Rural	Urban
**Academic/community**	Community	Community	Community	Academic
OT FTE*	2 x 0.5	1.0	1.0	2 x 1.0
**OT clinical experience**	> 15 years	< 5 years	< 5 years	> 20 years
	Paediatrics	General Rehab	General Rehab	Chronic Pain
	Older adults			General Rehab
**Rostered patients**	46 000	7 200	26 468	28 000
**Number of sites**	22	4	4	2
**OT onsite with physicians**	No	No	No	Yes
**EMR used across sites**	Yes	No (3 of 4)	No	Yes
**OT access to EMR**	Yes	Yes (3 of 4)	No	Yes
**EMR Use**	Referrals, charting, informal communication (messaging)	Referrals, charting, informal communication (messaging)	No access at time of study	Referrals, charting, informal communication (messaging)
**Patient charting**	EMR	EMR	Paper Files	EMR
**Referral process to OT**	Referrals through EMR and administered through central office.	Referrals received directly by OT through the EMR.	Referrals received to central administration by fax.	Referrals received directly by OT through the EMR.
**Referrals to OT**	Referrals made by physician. Other health providers may refer to occupational therapy; with physician notification through EMR.	Referrals can be made by any team member or patient self-referral.	Referrals made by physician. Other health providers may refer to occupational therapy, with physician notification.	Referrals can be made by any team member or patient self-referral.

*Full-time equivalency.

### Case 1: Very large rural community family health team

In case one the occupational therapists along with the interdisciplinary health providers and administrative staff were located in two buildings in the largest regional town, while the physicians worked in distributed clinics across the region. Despite the lack of co-location each key informant reported a strong sense of collaboration and connection. The EMR was the key structure for collaboration and integration of occupational therapy into the Family Health Team; face-to-face interaction with physicians is limited.

### Case 2: Small urban community family health team

Case two was a small Family Health Team with four separate sites located in a large urban setting with a culturally diverse patient population. The occupational therapist was located with nursing and other interdisciplinary health providers across the street from one of the main physician sites.

Lack of co-location was described as a key barrier in the integration of occupational therapy. The Family Health Team was planning a new building to house all team members.

### Case 3: Large rural family health team, one occupational therapist

Case three was a large rural Family Health Team providing primary care to approximately 45% of the local population. Having only been recently approved as a Family Health Team, the team was largely in the development phase. The Family Health Team had four separate sites. The occupational therapists and other interdisciplinary health providers were located at one site along with the administrative staff. Each site had its own EMR that could not communicate between sites. At the time of the study the occupational therapist did not have access to the EMR. The long-term goal was to move to one accessible EMR system.

### Case 4: Urban academic family health team

Case four was an urban academic Family Health Team with two sites; each with a full interprofessional complement of professions. Services were organized by interprofessional care teams, where patients were designated to a team of clinicians. Two full-time occupational therapists worked between the two sites. The Family Health Team was part of the university Department of Family Medicine and therefore had a dual objective of providing primary care services and training family medicine residents, along with an expectation of research.

### Cross case analysis

**Figure 1:**
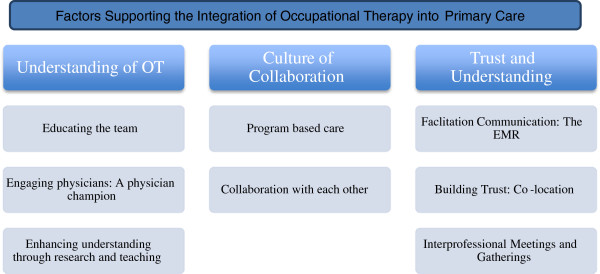
Themes and Subthemes.

Three main themes and eight subthemes were identified that influenced integration of occupational therapists into the Family Health Teams: understanding of occupational therapy, collaboration, communication and trust. See Figure 1 for visual outline of the themes and subthemes.

#### 1. Understanding occupational therapy

Fundamentally, an understanding of occupational therapy was critical and the tipping point for integration into the team. As referrals originated from team members, a basic understanding of the role of occupational therapy and patients who could benefit were required. Interdisciplinary health care providers and nurses described previous and current working relationships with occupational therapists, which in turn led to an understanding of the occupational therapy role within Family Health Teams.

The other integrated health professionals have been amazing. So I think they have a good idea of what OT is and I think a lot of them have worked with OT in the past (Occupational Therapist) 2P11:33:82

An understanding of and experience with occupational therapy in turn created a level of respect and natural integration into the team.

There’s a very healthy respect among our IHPs [interdisciplinary health providers] for the skill sets that they have and there’s a desire to include one another in the initiatives that they take on (Executive Director) 2P1:14:23

However, physicians had less direct day-to-day contact with occupational therapists, and less familiarity with the role of occupational therapy.

I feel that most family doctors didn’t and still don’t have a great understanding of the OT role (Physician) 4P4:1:6

Ultimately respondents felt that when team members had a good understanding of occupational therapy, referrals were made to the service.

That was the basis of our success here… that people really get what we do (Occupational Therapist). 1P1:93:220

Conversely, less familiarity with the role of occupational therapy was felt to result in an underutilization of services.

It’s underused, because I don’t think everyone knows what the OT can do (Nurse Practitioner) 2P5:5:13

### Educating the team

Occupational therapists across all sites used a number of strategies to educate physicians and team members about occupational therapy including formal presentations, educational rounds, ‘meet and greets’, information booths, brochures and information letters. Occupational therapists provided information about the profession, particularly, the services they currently offered within the Family Health Team along with examples of potential services that could be provided. All opportunities were seen as positive and contributing to an increased understanding of occupational therapy.

I’m working on trying to educate the team in what OTs can do (Occupational Therapist) 2P5:5:13

Promoting the role of occupational therapy was a particularly important element during the early integration into the team and a role that needed to be consciously adopted by occupational therapists.

### Engaging physicians: a physician champion

Physicians were seen as critical to the integration of occupational therapists as they were a key source of referrals. The identification of a physician lead, or physician liaison for occupational therapy was seen as an important strategy to enhance physician understanding and champion the occupational therapy discipline within the Family Health Team. Information from physician to physician was felt to have greater authority and credibility.

The communication was coming from a physician that they trust and he was saying ‘Use these services’ (Occupational Therapist) 1P1:94:221

A lack of physician engagement regarding the occupational therapy role was seen to significantly influence the integration of the role.

My regret about the occupational therapy program is that we haven’t done a good enough job of engaging the physician group in establishing that program … we’re definitely not utilizing her to the fullest extent that we could in her occupational specialization (Executive Director) 2P1:6:11

### Enhancing understanding through research and teaching

Team members at the academic Family Health Team had additional requirements to engage in both research and teaching activities. As a result, site four had a number of unique strategies that served to increase the understanding of occupational therapy and support a deeper integration into the team.

There are two absolutely primary mandates of clinical care and education and then obviously scholarly work … you can’t really separate clinical cases from education in this [Family Health Team]. So our nurses are doing so much of the clinical care and we are reviewing our teaching and the allied health group, including the OT’s, are absolutely woven into that. From co-bookings, to horizontal electives, to the more structured learning opportunities with the rounds, to working with different groups of the learners so family medicine residents and allied health workers sharing the case together. Some of the family residents teach the more junior learner and then going to an allied health person for some input. (Physician) 4P4:26:38

Training was a reciprocal and iterative activity; building an understanding of occupational therapy and supporting collaborative patient care.

Occupational therapists were expected to participate in interprofessional teaching rounds, one-on-one resident training, education clinics and occupational therapy student mentorship. Each activity offered an opportunity for the team to be exposed to the role of occupational therapy and work with the discipline.

One of the really helpful things that [the occupational therapists] did is to take some time at our interprofessional rounds and walk us through their vision in 6 months. Here are the types of cases that are getting referred, and here are success stories of why it was helpful to be involved. Here are some priority areas for us to think about. And that was again, a really nice diplomatic way of increasing our understanding. (Physician) 4P4:11:14

None of the other sites had formal structures in which to provide physician education, nor were they involved in any residency training.

A number of team members were involved in research with occupational therapists at the affiliated University and had been previously exposed to the role of occupational therapy in primary care. This research experience was felt to support the integration of the occupational therapist by offering a deeper understanding of the role.

I think we were better positioned already for a level, a deeper level of understanding of the role of OT and PT in primary care. (Physician) 4P4:3:8

Enhancing understanding through research cultivated opportunities to integrate occupational therapy into clinic programs.

I didn’t know much about chronic pain and [the OT] has been working in chronic pain for over 20 years so I was interested in being part of the research project and she has been mentoring me in that role so we have now created a new [pain] group (Social Worker) 4P2:25:43

#### 2. A culture of collaboration

While an understanding of occupational therapy facilitated referrals to occupational therapists, collaboration was seen as a benchmark of occupational therapy’s integration into Family Health Teams. Each site agreed that building team collaboration was a deliberate and intentional process.

We very deliberately, pretty much, do everything as a team with clinical work. (Physician) 4P4:22:36

Strong collaboration among interdisciplinary health providers was seen across all sites. In some cases assessments and interventions were conducted together with other interdisciplinary health providers.

*[Occupational therapist] and I have gone to a couple of home visits together; because the person was appropriate for my services and her services*. *(Social Worker) 3P9:20:74*

As many interdisciplinary health providers were also new to primary care they collaborated to support each other in their mutual integration into the team.

[the interdisciplinary health providers] … that’s my biggest source of support … so a lot of my referrals are actually coming from other allied health (Occupational Therapist) 3P11:33:82

Opportunities to collaborate at the point of care supported the integration of occupational therapy. However across sites there was notably less collaboration between the interdisciplinary health providers and physicians.

The physician group is not engaged strongly enough with the other health providers (Executive Director) 2P1:16:23

Less collaboration with physicians was attributed to a number of factors. First and foremost primary care has traditionally been practiced as a solo enterprise.

[The physicians] have always been the general practitioner that has done everything for their patients (Executive Director) 3P7:41:104

There was a sense that interprofessional collaboration may diminish the physicians’ sense of control.

I am sure there are a lot of physicians that do not like the ball being taken from them (Physician) 1P5:16:53

As physicians could see the benefit of occupational therapy services, opportunities for collaboration would be enhanced.

As physicians refer to the occupational therapist and have comfort level in what they’re getting back, that [occupational therapy] will improve [patient care]. More referrals will come and there will be more of an interaction. (Physician) 3P10:55:22

As the shift to interprofessional teams was relatively new, it was also felt that physicians were not used to having access to so many resources and needed to gain comfort with a team based approach

They’re not used to having this type of resources available to them on a daily basis in their clinics (Executive Director) 3P7:49:118

### Program based care

Each site offered a number of health promotion and chronic disease management and prevention programs ranging from mental health, falls prevention, chronic pain and diabetes management. Aligning occupational therapy services within current programs of care provided an opportunity to integrate into the team.

There’s a COPD group that’s held here and I provide some consultation to that group and I’m slowly tying to integrate myself into some other groups we’re going to be starting (Occupational Therapist) 1P1:4:9

Integration into programs occurred in a number of ways. In some cases occupational therapists noted a gap in program offerings, which led to the development of a new program. More frequently, occupational therapists or other team members identified programs that had high volume or wait lists that would benefit from an occupational therapy perspective.

Our program is really busy .. it’s great to have that opportunity to put that person with [the occupational therapist] that specializes and might be able to have the time to do it (Social Worker) 3P9:38:86

The program focus also provided new opportunities to collaborate and engage in program development.

One of our ideas is to have a caregiver stress program … that was going to be a collaboration between [occupational therapist] and myself and the mental health therapist (Social Worker) 3P9:31:76

At two of the sites physicians were aligned with specific programs, which provided a formal opportunity to connect with physicians.

### Collaborating with each other

As essential as interprofessional collaboration was in supporting the integration of occupational therapy, the ability to collaborate with occupational therapy colleagues both within and outside of the Family Health Team was also important. Occupational therapists shared resources, engaged in clinical consultations, and provided strategies to each other to support integration into the team.

This whole group of occupational therapists [working in FHT’s] are pioneers in the OT role. So any way we can support one another (Occupational Therapist) 1P3:62:225

Having two occupational therapists at one Family Health Team was seen to facilitate the integration of the role in number of ways. Most importantly it provided professional support and confidence to try new roles and share ideas. Simply having two individuals increased exposure to occupational therapy within the Family Health Team and enhanced the professional profile.

To have each other … I can’t imagine doing this role … as one person (Occupational Therapist) 1P1:27:54

#### 3. Communication and trust

Communication and trust were essential components of collaboration and the integration of occupational therapy, and were supported by a number of strategies including co-location, EMR and formal and informal meetings and gatherings.

### Facilitating communication: the electronic medical record

A single and accessible EMR was a critical feature in supporting the integration of occupational therapy into Family Health Teams. The EMR enabled both formal and informal communication with physicians and other team members through the messaging system and patient records. The instant messaging function served as an internal communication system.

I think the EMR allows us to communicate effectively. We can instant message and that piece provides opportunity (Social Worker) 1P2:24:65

The EMR provided a means to collaborate when co-location of team members was not possible, supporting virtual interprofessional teams.

The EMR is fabulous because not only can you communicate back and forth, but everyone can see everyone’s charts. It is like one big family medicine chart. (Physician) 1P5:12:41

The EMR was also seen to support efficient and informed clinical practice.

The OT gets a snapshot of that patient and they’re better equipped to do what they need to do. And to get to the point a lot quicker (Physician) 1P3:40:127

### Building trust: co-location

While an integrated EMR provided a foundation for communication, the opportunity for team members to connect face-to-face was pivotal in developing relationships and supporting the integration of occupational therapy. The importance of occupational therapy being located with the entire team cannot be underestimated. Only one of the four cases had a full interprofessional team located in the same building, however two of the other cases had plans to consolidate their clinics. Co-location offered opportunities for occupational therapists to engage in informal communication, have ‘hallway consults’ and be visually present; all of which contributed to understanding the OT role and building of trust.

*There are other times where you are not sure if [occupational therapy] would be helpful or not. It is much more relevant to have an [informal] case conversation first and then whatever you end up writing in [the EMR] references the conversation, which is obviously much richer. (Physician*) *3P4:15:22*

One site created team rooms where all team members worked in a common desk area, along with common lunch rooms and meeting spaces. When co-location occurred only with other interdisciplinary health providers and nurses, the benefits of communication and understanding were also identified; however as physicians were a key source of referrals their physical presence was viewed as a critical.

Physically we don’t see the [physicians] very often. I think that can spark some reminders, or spark some ideas, as well as is great for relationship building. (Social Worker) 4P9:45:110

### Interprofessional meetings and gatherings

Formal meetings provided opportunities for team members to interact, most notably in cases where occupational therapists were off-site from physicians.

Just going to the meeting is an opportunity to talk, see what everyone does (Occupational Therapist) 1P1:66:143

Just as important as meetings, social gatherings supported team building and enabled the team to get to know each other as individuals.

We’ve spent some good networking sessions … you get to know that person and all of a sudden “OK, I’ll trust you with my patient” (Executive Director) 1P3:19:73

Ultimately, the integration of occupational therapy into the primary care teams was grounded in three key factors: trust, understanding, and communication. Meetings and gatherings provided opportunities to facilitate connections and team building.

## Discussion

Integration has been described as one end of the continuum that extends from complete autonomy and independence at one extreme to complete integration of professional services at the other [25]. In this study, the integration of occupational therapists was observed to range along this continuum and varying both between and within the Family Health Teams. In these cases, occupational therapists were more integrated with the other interdisciplinary health providers such as social workers and pharmacists, than with either nurses or physicians. Vertical and horizontal integration have been used to describe the integration of health services. Horizontal integration refers to the grouping of similar organizations or services, while vertical integration “services a network of organizations that provides or arranges to provide a coordinated continuum of services to a defined community” [26]. Within the Family Health Teams occupational therapists tended to work closely and collaborate with other allied health professionals in the delivery of health services. Allied health professionals had a common goal of supporting the physicians in the delivery of primary care. While each had different disciplinary perspectives, occupational therapists could be described as being horizontally integrated with their allied health counterparts. Each was remunerated in a similar fashion, worked in close physical proximity, had informal communication structures and provided some degree of collaborative patient care.

In contrast, occupational therapists had relatively little direct contact and few interactions with physicians. The occupational therapy role was seen as supporting the continuum of health services within the Family Health Team and integration could be envisioned as being vertical relative to the physicians. This is congruent with the literature reporting that a key barrier in the implementation of interprofessional teams has been the hierarchical structures within primary care [27-29]. Of note, however is a recent study suggesting that younger cohorts of male physicians are more likely to collaborate with occupational therapists, and other health professionals than older counterparts or younger female physicians [30]. Occupational therapists at the academic site experienced a high level of integration into the team, including with physicians, nurses and other interdisciplinary health providers. Given the focus on collaboration and teamwork in the training of family medicine practitioners, it makes sense that younger physicians who have had experience with interprofessional collaboration enact this as practicing physicians.

This study also found that the extent of occupational therapists integration into Family Health Teams was influenced by the nature of services provided. Integration was more fully realized within chronic and complex disease programs of care, such as a diabetes or seniors program, than one- time referrals to occupational therapy. This study suggests a plausible explanation for this phenomenon. The more structured programs served to identify and formalize a team of providers and offered an opportunity to develop common patient goals and a shared vision of service delivery. This in turn facilitated communication and the implementation of processes to support the programs, such as meetings and common program outcomes. Russell and colleagues [31] examined chronic disease management programs and found that organizational features had the greatest influence on patient outcomes. In particular, those clinics with the presence of a nurse practitioner had better outcomes and high-quality chronic disease management care was found most commonly in clinics with an interprofessional team. The success of chronic disease management programs in part contributed to the collaborative nature of the care, highlighting the importance and benefit of integrating professionals within programs of care.

At the same time it is recognized that not all care provided by occupational therapists within primary care teams will be program based. Leutz [32] described five laws for integration, one of which was “you can integrate all of the services for some of the people, some of the services for all of the people, but you can’t integrate all of the services for all of the people” (p. 83). This may hold true for occupational therapists in the sense that certain elements of their work within the teams may be more individual and consultative in nature.

The literature has described a number of factors that support interprofessional teamwork in primary care [21,28,29]. Xyrichis and Lowton [21] identified both team structures and team processes that support collaboration. As was seen in this study, Family Health Teams with a greater number of structures to support teamwork had occupational therapists that were more fully integrated. Processes that were seen to support the integration of occupational therapist included co-location, a common EMR, formal and informal communication structures and team meetings. Each of these processes naturally facilitated the integration of occupational therapy into the team by building trust, understanding and familiarity. It was the processes and structures, more than the personal characteristics of the occupational therapist that appeared to influence integration. However, the two sites with the greatest supports also had occupational therapists with substantial work experience. Further research is required to explore the relationship between personal characteristics and the integration process. A recent study [22] examined teamwork within twenty-one Family Health Teams in Ontario, Canada. A survey was used to identify organizational factors contributing to the functioning of an interprofessional primary care team. The study found that culture, leadership and EMR functionality predicted team climate. Each of these elements was also seen to support the integration of occupational therapy in this study.

Studies examining the integration of pharmacists reported some lack of understanding of the role of the pharmacist, but not to the extent found in this current study [13-15]. It is not surprising that the lack of understanding about a profession’s role impedes their integration into the team. The current siloed approach to the training of health care practitioners and practice of health care may be a contributor [33]. For disciplines new to primary care, there will be a natural learning curve about both the roles of other professionals as well as their own role in a new practice setting. Kolodziejak and colleagues [15] outlined a step-by-step process to support the integration of pharmacists into established primary care teams. Part of the process of integration included defining the role prior to joining a team and determining early credibility. The current study found a number of intentional strategies were used to integrate occupational therapy within the team, however more formal guidelines to Family Health Teams who have new professionals could further support integration.

The study also found that informal and formal support by occupational therapy colleagues was also helpful in supporting integration. Communities of practice have been shown to support knowledge translation [34,35] and this could be another intentional strategy that is enacted.

Interprofessional education occurs “when two or more professions learn with, from and about each other to improve collaboration and the quality of care*”*[36]. In the case of the academic Family Health Team, the educational processes designed to support physician learning provided a natural opportunity and environment to educate team members of their roles. Without such structures, the occupational therapists at the other sites did not have a forum to provide formal physician education. A growing amount of literature on interprofessional education suggests that experiential based learning is an effective strategy to teach health professionals the competencies of collaborative practice [37,38]. While there are only a small number of academic Family Health Teams, there is much to be learned about the research and teaching activities that can support the integration of new team members.

It must be remembered that this study was limited to four sites. Given the influence of structures and processes on collaboration and integration, it is anticipated that additional sites might have provided further insights into the variety of other assets or constraints to interprofessional integration. Occupational therapy is a new profession within Family Health Teams and the paper focuses on the early integration in the team. Therefore the integration of occupational therapy will continue to evolve and be shaped by individual, team and organizational development. The study was exploratory in nature and while it provides insights into the emerging role of occupational therapy within a primary care context, the results cannot be broadly generalized.

This study builds the foundation for further research. A longitudinal study would provide insights into how health professionals are integrated into teams over time. It would also be of value to understand how integration influences health outcomes and more specifically to use a framework of systems integration in which to understand interprofessional primary care teams. Finally, it would be important to explore how professionals within Family Health Teams were integrated into the broader community services.

## Conclusions

With an increased emphasis on interprofessional primary care, new professions will continue to be integrated into primary care teams. Based on the current study the following strategies and structures should be considered to support occupational therapists entering primary care teams.

1. Occupational therapists entering primary care need to formally include the education of team members in their professional role. Education on the role of occupational therapy and services provided needs to be directed to all team members, with specific focus on physicians.

2. Occupational therapists need to ensure they gain full access to the EMR to support both informal communication, through the internal messaging features, as well as formal patient documentation and referrals.

3. Occupational therapy fieldwork placements can provide a mechanism to engage the team in learning about other professions. Student occupational therapists should also be involved in the education of team members.

4. When possible, occupational therapists should actively participate in educating students from other health disciplines, including offering shadowing opportunities, providing handouts, arranging co-bookings or developing inservices.

5. Occupational therapists need to actively develop their role in existing interprofessional groups and programs offered within the primary care setting. Working within a structured program provides an opportunity to work closely with team members and can facilitate a deeper understanding of the occupational therapy.

6. Occupational therapists need to attend networking events, meetings, inservices and social functions to build relationships with team members.

The study adds to the growing body of literature that has identified structures and processes to support interprofessional collaboration in primary healthcare. Exploring the integration of an emerging discipline in primary care underscores the necessity of ensuring team members have an understanding of the roles and scope of each team member. The study also highlights the critical role that communication structures, such as formalized meetings and EMR’s, have in supporting the integration of new professions.

## Competing interests

The authors declare that they have no competing interests

## Authors’ contributions

CD, LL, CB, CC contributed to the design of the study. CD participated in the coordination and acquisition of data. CD, LL, CB contributed to the analyses and interpretation of data. CD participated in the draft of the manuscript and LL, CB, CC provided feedback and approval of the final draft. All authors read and approved the final manuscript.

## Pre-publication history

The pre-publication history for this paper can be accessed here:

http://www.biomedcentral.com/1471-2296/14/60/prepub

## References

[B1] Letts LJ Optimal positioning of occupational therapy Can J Occup Ther 2011 78 209 219 10.2182/cjot.2011.78.4.2 22043552

[B2] Howey M Angelucci T Johnston D Townsend E Occupation-based program development in primary health care OT Now 2003 11 3 4 7

[B3] Clark F Azen SP Zemke R Jackson J Carlson M Mandel D Occupational therapy for independent-living older adults: A randomized controlled trial JAMA 1997 278 1321 1326 10.1001/jama.1997.03550160041036 9343462

[B4] Richardson J Letts L Chan D Stratford P Hand C Price D Hilts L Coman L Edwards M Baptiste S Law M Rehabilitation in a primary care setting for persons with chronic illness: A randomized controlled trial Prim Care Res Devo 2010 11 382 395 10.1017/S1463423610000113

[B5] Klaiman D Increasing access to occupational therapy in primary health care OT Now 2004 6 1 14 1

[B6] McColl MA Shortt S Godwin M Smith K Rowe K O’Brien P Donnelly C Models for integrating rehabilitation and primary care: A scoping study Arch Phys Med Rehab 2009 90 523 531 10.1016/j.apmr.2009.03.017 19735780

[B7] Tse S Penman M Simms F Literature review: Occupational therapy and primary health care NZ J Occup Ther 2003 50 17 23

[B8] Health Canada First Minister’s Meeting on the Future of Health Care 2004: A 10-year plan to strengthen health care 2004 http://www.hc-sc.gc.ca/hcs-sss/delivery-prestation/fptcollab/2004-fmm-rpm/index-eng.php

[B9] Hutchinson B Levesque JF Strumpe E Coyle N Primary health care in Canada: Systems in motion MilBank Q 2011 89 256 288 10.1111/j.1468-0009.2011.00628.x 21676023 PMC3142339

[B10] Ontario Society of Occupational Therapists Occupational therapy and Family Health Teams http://www.osot.on.ca/eng/otinont/familyHealthTeams.asp

[B11] Belle Brown J Lewis L Ellis K Beckhoff C Stewart M Freeman T Kasperski MJ Sustaining primary health care teams: What is needed J Interprof Care 2010 24 463 465 10.3109/13561820903417608 20441398

[B12] Sargeant J Loney E Murphy G Effective interprofessional teams: Contact is not enough to build a team J Contin Educ Health Prof 2008 28 228 234 10.1002/chp.189 19058243

[B13] Bradley F Elvey R Ashcroft DM Hassell K Kendall J Sibbald B Noyce P The challenge of integrating community pharmacists into the primary health care team: A case study of local pharmaceutical services (LPS) pilots and interprofessional collaboration J Interprof Care 2008 22 387 398 10.1080/13561820802137005 18800280

[B14] Dolovich L Pottie K Kaczorowski J Farrell B Austin Z Rodriguez C Gaebel K Sellors C Integrating family medicine and pharmacy to advance primay care therapeutics Clin Pharmacol Ther 2008 83 913 917 10.1038/clpt.2008.29 18388882

[B15] Kolodziejak L Remillard A Neubauer S Integration of a primary healthcare pharmacist J Interprof Care 2010 24 274 284 10.3109/13561820903130149 20388026

[B16] Drummond D Commission on the Reform of Ontario’s Public Services: Public Service for Ontarians: A Path to sustainability and excellence 2012 Toronto, ON: Queen’s Printer for Ontario

[B17] Standing Senate Committee on Social Affairs, Science and Technology Time for Transformative Change: A review of the 2004 Health Accord 2012

[B18] Yin RK Case Study Research: Design and Methods 2009 3 Thousand Oaks California: SAGE Publications Inc

[B19] Stake R The Art of Case Study Research 1995 Thousand Oaks, California: SAGE Publications Inc

[B20] Salminen AL Harra T Lautama T Conducting case study research in occupational therapy Aus Occup Ther J 2006 53 3 8

[B21] Xyrichis A Lowton K What fosters or prevents interprofessional teamworking in primary and community care? A literature review Int J Nurs Stud 2008 45 140 153 10.1016/j.ijnurstu.2007.01.015 17383655

[B22] Howard M Brazil K Akhtar-Danesh N Agarwal G Self-reported teamwork in family health team practices in Ontario Can Fam Phys 2011 57 May e185 e191 PMC3093607 21571706

[B23] Krefting L Rigor in qualitative research: The assessment of trusworthiness Am J Occup Ther 1991 43 214 222 2031523 10.5014/ajot.45.3.214

[B24] Golafshani N Understanding reliability and validity in qualitative research Qual Report 2003 8 597 607

[B25] Glendinning C Breaking down barriers: Integrating health and care services for older people in England Health Policy 2003 65 139 151 10.1016/S0168-8510(02)00205-1 12849913

[B26] Devers KJ Shortell SM Gilles RR Anderson DA Mitchell Erickson K Implementing organized delivery systems: An integration scorecard Health Care Manage Rev 1994 19 3 7 30 7822193

[B27] Craigie FC Hobbs RF Exploring the organizational culture of exemplary community health center practices Fam Med 2004 36 733 738 15531989

[B28] Poulton BC West MA The determinants of effectiveness in primary health care teams J Interprof Care 1999 13 7 18 10.3109/13561829909025531

[B29] Shaw A De Lusignan S Rowlands G Do primary care professionals work as a team: A qualitative study J of Interprof Care 2005 19 396 405 10.1080/13561820500053454 16076600

[B30] Sarma S Devlin RA Thind A Chu MK Canadian family physicians’ decision to collaborate: Age, period and cohort effects Soc Sci Med 2012 75 1811 1819 10.1016/j.socscimed.2012.07.028 22898720

[B31] Russell GM Dabrouge S Hogg W Geneau R Muldoon L Tuna M Managing chronic pain in Ontario primary care: The impact of organizational factors Ann Fam Med 2009 7 309 318 10.1370/afm.982 19597168 PMC2713154

[B32] Leutz WN Five laws for integrating medical and social services: Lessons from the United States and the United Kingdom Milbank Q 1999 77 77 110 10.1111/1468-0009.00125 10197028 PMC2751110

[B33] Pecukonis E Doyle O Bliss DL Reducing barriers to interprofessional training: promoting interprofessional cultural competence J Interprof Care 2008 22 417 428 10.1080/13561820802190442 18800282

[B34] Barwick MA Peters J Boydell K Getting to update: Do communities of practices support the implementation of evidence-based practice? J Can Acad Child Adolesc Psych 2009 18 16 29 PMC2651208 19270845

[B35] Li LC Grimshaw JM Nielsen C Judd M Coyte PC Graham ID Use of communities of practice in business and health care sectors: A systematic review Implement Sci 2009 4 27 http://www.implementationscience.com/content/4/1/27 10.1186/1748-5908-4-27 19445723 PMC2694761

[B36] CAIPE Defining IPE http://www.caipe.org.uk/resources/defining-ipe/

[B37] Payler J Meyer E Humphris D Pedagogy for IP education – what do we know and how can we evaluate it? Learn Health Soc Care 2008 7 64 78 10.1111/j.1473-6861.2008.00175.x

[B38] Sargeant J Theories to aid understanding and implementation of IP education J Contin Educ Health Prof 2009 29 178 184 10.1002/chp.20033 19728383

